# Spontaneous expulsion of an intrabronchial sharp metallic foreign body and migration to the gastrointestinal tract at Muhimbili National Hospital: Case report and literature review

**DOI:** 10.1016/j.ijscr.2020.05.100

**Published:** 2020-06-12

**Authors:** Zephania Saitabau Abraham, Aveline Aloyce Kahinga, Kassim Babu Mapondella, Enica Richard Massawe, Daudi Ntunaguzi

**Affiliations:** a Department of Surgery, University of Dodoma, College of Health and Allied Sciences, Box 259, Dodoma, Tanzania; b Department of Otorhinolaryngology, Muhimbili University of Health and Allied Sciences, Box 65001, Dar es Salaam, Tanzania

**Keywords:** Spontaneous, Expulsion, Intrabronchial, Foreign body, Tanzania

## Abstract

**Introduction:**

Aspirated foreign bodies continue to pose challenges to Otorhinolaryngologists and are potentially life threatening thus an otorhinolaryngological emergency. The main stay of treatment of foreign bodies (FBs) in the tracheobronchial tree remains to be bronchoscopy while bearing in mind earlier and safer removal of such foreign bodies. Spontaneous expulsion of an Intrabronchial foreign body is a rare entity with few cases reported in the available literatures.

**Presentation of case:**

We are reporting a rare case of a 3-year old male child who presented to otorhinolaryngology department with a 2-days history of foreign body inhalation (sharp metallic pin) prior admission which undergone spontaneous migration and went to be excreted in feaces after passing through the gastrointestinal tract.

**Discussion:**

It is unwise, dangerous and inadvisable to wait for spontaneous expulsion in cases of intrabronchial foreign body but while preparing for endoscopy, a constant watch should be kept over the patient and every forceful bout of cough should be looked with suspicion of spontaneous expulsion and a danger of foreign body lodgment into subglottis during such rare but possible occurrence should be kept in mind.

**Conclusion:**

It’s always worth to rule out the possibility of foreign body inhalation in children with sudden onset of difficulty in breathing to avoid diagnostic delays.

## Highlights

• Early intervention of inhaled foreign bodies should be advocated.
• Spontaneous migration of foreign bodies may be hazardous to both respiratory and digestive tracts.
• Children with a history of sudden onset of difficulty in breathing must be considered to have aspirated foreign body(ies) until proven otherwise.
• Imaging remains to be of paramount importance when investigating children suspected to have inhaled foreign body(ies).

## 1 Introduction

Foreign body inhalation in the tracheobronchial tree is a dangerous and life threatening condition with adverse outcomes [1]. Spontaneous expulsion and consequent ingestion of an intrathoracic metallic foreign body is a very rare event reported in literatures thus warranting it to be reported once encountered in daily clinical practice. Spontaneous expulsion should neither be expected nor experienced; thus, early removal with a bronchoscope should be performed to prevent complications rather than awaiting spontaneous complications [[1], [2], [3]].

Children, and in particular the very young, are the most vulnerable for aspiration and ingestion of FBs. Although the spectrum of FBs varies from country to country, depending on the diet and customs of the population, the most common foreign body aspirated causing injuries are from diminutive food items [[4], [5], [6]]. Such occurrence has also been observed at our local hospital setting where foreign bodies aspirated are prominently food items.

The mechanism of migration of FBs may be due to high expiratory flow rate generated during coughing. This initial peak of expiratory flow lasts about 30–50 milliseconds and may reach rates as great as 12 L/s [7]. Shape and chemical nature of the foreign body also play a role in its migration. Irregularly shaped and sharp pointed objects are less likely to migrate since they easily stick to the mucosa. Inorganic FBs are usually inert and evoke less inflammatory response even if they migrate. Organic foreign bodies can cause severe inflammatory response and with fluid absorption, they can increase in size and may cause airway obstruction [8].

The possibility of migration of a foreign body should always be kept in mind and dormia basket is a safe alternative to conventional method [9]. Spontaneous expulsion of foreign bodies in the airways occurs even more rarely in approximately 1–2% of cases [1,10,11].

The main stay of treatment of foreign bodies in the airways remains to be endoscopic removal and this should be done earlier and safely to prevent potential complications [[1], [2], [3],[10], [11], [12]].

We report an unusual case of 3-year old child who expelled in stool an Intrabronchial sharp metallic foreign body which was ingested into the digestive tract following spontaneous explosive bouts of cough. This work has been reported in line with the SCARE criteria [13].

## 2 Presentation of case

**Fig. 1:**
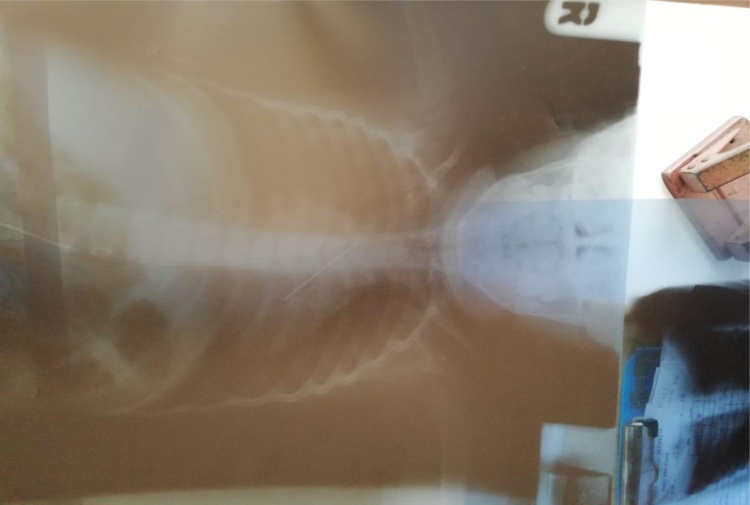
Chest x-ray showing the metallic pin being located in the left main bronchus.

The 3-year old male child presented to otorhinolaryngology clinic after being referred from one of the peripheral health facility following inhalation of sharp metallic pin two-days prior admission followed by cough and difficulty in breathing and a state of restlessness. Chest X-ray was ordered which revealed the presence of the metallic pin in the left main bronchus contrary to the usually expected experience where majority of foreign bodies lodge in the right main bronchus due to anatomical differences between the left and right main bronchus (Fig. 1).

**Fig. 2:**
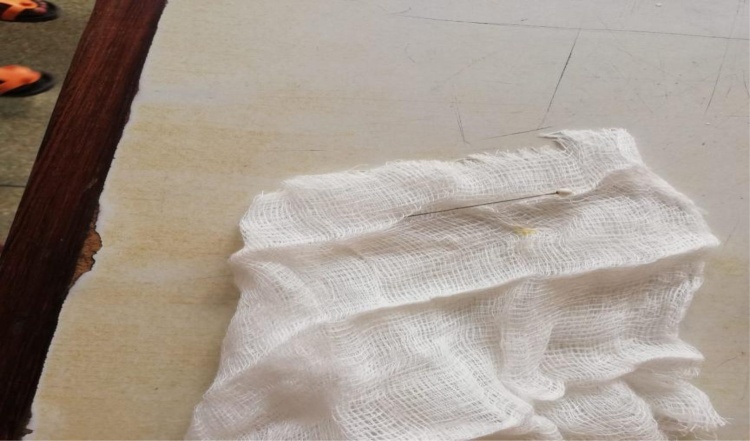
Showing the pin after being passed in stool.

Emergency bronchoscopy was planned on the same day of admission and intraoperatively only the sharp end of the metallic pin could be visualized in the left main bronchus while the other end with a plastic knob couldn’t be visualized and several intraoperative attempts of removing the foreign body ended with vain and such metallic pin couldn’t be extracted from the bronchus due to barrier impact exerted by the unseen knob intraoperatively. The procedure was postponed and a second-look bronchoscopy was planned to be done on the following day but on the same day, his mother reported that the child had several spontaneous bouts of cough which ended up with expectoration of the metallic pin and she reported it to have seen at the oropharynx but it was swallowed by the child. The child was kept under observation with planned control chest x-ray but on the following day post the event of swallowing the metallic pin, the pin was passed in stool by the child (Fig. 2). The child was then kept on intravenous dexamethasone 4 mg administered 8-hourly for 24 h, intravenous ceftriaxone 500 mg given 12 hourly for 24 h and intravenous paracetamol 300 mg administered 8-hourly for 2 h and then switched oral cefixime administered 5mls once daily for 7 days.

The child was further kept under observation for 48 h after passing the pin in stool before she was discharged home.

## 3 Discussion and conclusion

Foreign body inhalation has been a subject to be emphasized in clinical practice due to associated potential mortality if no immediate intervention is conducted. Children account for the vast majority of all foreign body aspirations reported. Foreign bodies can either be organic (eg, peanuts, peas) or inorganic (eg, plastic caps, pins, screws, nails, teeth). Clinically, patients may present either with acute respiratory failure requiring urgent intervention or with recent onset of respiratory symptoms, including breathlessness, wheezing, coughing, and expectoration [2,3,5,6,11].

In some rare occasions, an expelled foreign body from one part of the airway can be swallowed or migrate to another location. Several cases of patients who have swallowed a foreign body into their digestive tract after expectoration have been reported [1,6,14]. Although most ingested foreign bodies pass through the gastrointestinal tract uneventfully over a period of seven to ten days, large, sharp, or pointed objects can cause other complications, such as perforation, obstruction, or concealed hemorrhage [14].

Usually, there is a suggestive history of choking, although the classic clinical presentation, with coughing, wheezing, and diminished air inflow, is seen in less than 40% of the patients [15,16]. Other symptoms include cyanoses, fever, and stridor. Sometimes, foreign body aspiration (FBA) can be completely asymptomatic [5,17].

As subglottis is the narrowest part in the upper respiratory tract in children, there is always an obvious risk during spontaneous expulsion of foreign body from tracheobronchial tree, the foreign body may lodge into subglottis and may impose a life threatening emergency requiring urgent intervention [1,7].

Removal of intrabronchial foreign bodies should be performed once the diagnosis is made. This can be achieved either by bronchoscopy or thoracotomy. Rigid bronchoscopy is the gold standard for removal of foreign bodies from tracheobronchial tree under direct vision [1,[4], [5], [6],^8^,^14^].

At our hospital, foreign body aspiration is one of the otorhinolaryngological emergencies and rigid bronchoscopy has been the modality of removing foreign bodies from the airways similar to the first attempt made in removing the metallic pin as it has been well narrated in this case report.

This case report thus highlights the importance of early intervention of foreign body inhalation through rigid bronchoscopy rather than awaiting spontaneous expectoration which may be hazardous to both the respiratory and digestive tracts through perforation and even concealed hemorrhage which are disastrous to paediatric patients bearing in mind the sharp metallic pin which has been reported in this case report.

## Declaration of Competing Interest

On behalf of all authors, we would like to declare that we DO NOT HAVE conflicts of interest associated with this manuscript and this is in terms of employment, consultancies, stock ownership, honoraria, grants or any source of funding.

## Source of funding

We DO NOT have any funding source for this case report.

## Ethical approval

Ethical approval was obtained from Researh and Ethics Committee of Muhimbili National Hospital MNH/Vol1/2018.

## Consent

Written informed consent was obtained from the legal parent of the child for publication of this case report and accompanying images. A copy of the written consent is available for review by the Editor in Chief of this journal on request.

No patients’ initials have not been included for privacy.

## Author contribution

ZSA, DN, ERM, AAK and KBM; Participated in design and manuscript writing, ZSA; Corresponding author; ZSA, DN, ERM, AAK and KBM; Read and approved the final draft.

## Registration of research studies

NA.

## Guarantor

Dr. Zephania Saitabau Abraham and Dr. Daudi Ntunaguzi accept full responsibility for the study and guarantee its accuracy.

## Provenance and peer review

Not commissioned, externally peer reviewed.
